# Stochastic Difference-Dedicated Configuration Interaction for Magnetic Exchange in Large Active Spaces

**DOI:** 10.1021/acs.jctc.6c00620

**Published:** 2026-06-16

**Authors:** Luca Bonfirraro, Oskar Weser, Carmen J. Calzado, Vincent Robert, Giovanni Li Manni

**Affiliations:** † 28326 Max Planck Institute for Solid State Research, Heisenbergstr. 1, Stuttgart 70569, Germany; ‡ Departamento de Química Física, 16778 Universidad de Sevilla, c/Profesor García González, s/n., Sevilla 41012, Spain; § Laboratoire de Chimie Quantique de Strasbourg, Institut de Chimie de Strasbourg, 27083 CNRS/Université de Strasbourg, 4 rue Blaise Pascal, Strasbourg 67000, France

## Abstract

The simulation of magnetic properties in strongly correlated systems remains a central challenge in electronic structure theory. The Difference-Dedicated Configuration Interaction (DDCI) method is widely regarded as a gold standard for computing magnetic exchange couplings, but its applicability is limited to small magnetic systems due to the steep growth of the configuration-interaction space with the number of correlated electrons and orbitals. Here, we introduce a stochastic formulation of DDCI based on Full Configuration Interaction Quantum Monte Carlo (FCIQMC) and the Generalized Active Space framework, which largely alleviates the computational bottleneck of conventional DDCI. The implementation is validated by comparison with conventional DDCI for the spin ladder of a trinuclear [Mn­(IV)_3_O_4_]^3+^ cluster (*S*_local_ = 3/2). Using a small CAS­(9,9) reference, DDCI fails to reproduce the spin-state energy differences defined by a two-parameter (*J* = −76, *J*′ = −11 cm^–1^) Heisenberg–Dirac–van Vleck Hamiltonian extracted from experimental measurements. In contrast, Stochastic-DDCI enables calculations with a much larger CAS­(33,21) reference and reproduces the experimental spin ladder with remarkable accuracy, yielding deviations smaller than 33 cm^–1^ with respect to the spin ladder extracted from the experimental measurements. This development extends DDCI methodologies to substantially larger active spaces and opens the door to the study of more complex magnetic systems.

## Graphical abstract

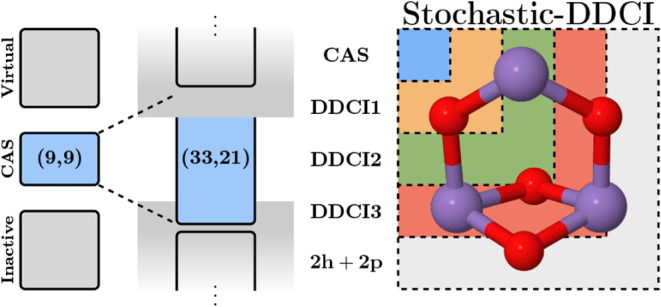

## 1 Introduction

Polynuclear transition metal (PNTM) clusters represent a wide class of strongly correlated systems exhibiting many low-energy spin states that play a pivotal role in catalysis and magnetism and provide valuable insights for the design of biomimetic catalysts of industrial relevance. A wide variety of systems has been reported in the literature, ranging from molecular compounds^1,2^ to extended materials.^3,4^ The physical properties of these compounds are generally attributed to the presence of many unpaired electrons. Their highly correlated nature may give rise to unusual phenomena such as magnetic bistability in single-molecule magnets^5–7^ and superconductivity.^8–10^

Despite their ability to address large systems, conventional density-functional theory methods are often inadequate for systems with many unpaired electrons, particularly those characterized by noncollinear ground states,^11^ and are therefore rarely applied to magnetic systems with spins *S*_local_ > 1/2.^12^ To address these challenges, Caballol and co-workers^13^ rigorously analyzed the broken-symmetry approach, in which spin-contaminated single-determinant states provide estimates of magnetic exchange couplings. This strategy is not limited to molecular systems and has offered key insights into extended cuprate materials.^14,15^ In contrast, wave function-based methods naturally yield pure spin states, providing energies and eigenstates suitable for benchmarking spin-only Hamiltonian models. Magnetic interactions are typically described using the Heisenberg–Dirac–van Vleck (HDvV) Hamiltonian, which introduces exchange coupling constants *J*_*ij*_ between local spins *Ŝ*_*i*_,

1 $$Ĥ=-2\underset{ij}{\sum }J_{ij}{Ŝ}_{i}\cdot {Ŝ}_{j}$$

More sophisticated spin Hamiltonians extend this model to include single-ion anisotropies, Dzyaloshinskii–Moriya interactions, and anisotropic exchange,^16,17^ which originate from first- and second-order spin–orbit couplings.

In modern electronic structure theory, a number of wave function based strategies exist that are able to tackle large active space wave function. These include Density Matrix Renormalization Group (DMRG),^18–35^ Selected-CI,^36–51^ Generalized Active Space,^52^ Localized Active Space,^53–58^ Full Configuration Interaction Quantum Monte Carlo (FCIQMC) in its original form^59–65^ and related stochastic methods such as Stochastic-CASSCF,^66,67^ Stochastic-GASSCF,^68,69^ and the perturbatively corrected single-CSF-SCF method published very recently.^70^ While methods to solve large active spaces are nowadays reaching a mature status, strategies that can capture weaker form of electron correlation (dynamic correlation) beyond large active spaces are rare. Few examples include DMRG/FCIQMC-based CASPT2/NEVPT2,^71–81^ Driven Similarity Renormalization Group (DSRG),^82–86^ and Multiconfiguration pair-density functional theory.^87,88^

Among wave function-based methods, the Difference-Dedicated Configuration Interaction (DDCI) method^89^ is renowned for providing highly accurate energy differences, often within tens of wavenumbers, in spin-coupled systems.^90^ DDCI, however, like other conventional active-space-based methods, suffers from steep scaling with the number of correlated electrons and orbitals, limiting its application to systems with few magnetic centers (bi-, tri-, and tetra-nuclear complexes), typically with local spins *S*_local_ = 1/2 or 1.^91–93^ The largest system studied with DDCI to date is a ferromagnetic copper­(II) [3 × 3] grid (*S*_local_ = 1/2) using a Complete Active Space, CAS­(9,9), reference space.^93^ Building on Stochastic-CASSCF^66^ and recent stochastic advances, including stochastic Generalized Active Space^69^ and Stochastic Perturbation Theory strategies,^72,81^ we have formulated DDCI in a stochastic framework, Stochastic-DDCI, capable of handling much larger active spaces. This development overcomes the CAS­(9,9) barrier, providing the required efficiency and scalability to tackle more complex systems, including PNTM clusters. Algorithmic details and the applicability of Stochastic-DDCI are introduced in this work.

Its performance is illustrated on the low-lying electronic spectrum of a triangular manganese­(IV) complex, [Mn_3_O_4_(OH)­(bpea)_3_]­(ClO_4_)_3_, from here on simply referred to as [Mn­(IV)_3_O_4_]^3+^ (Figure 1).^94,95^ The method is first validated by reproducing established nonstochastic DDCI results using a minimal CAS­(9,9) reference via the original CASDI code.^96^ Building on this, Stochastic-DDCI enables a considerable expansion of the reference space to CAS­(33,21), generating DDCI spaces previously inaccessible to conventional implementations and demonstrating its power to tackle complex, highly correlated PNTM systems.

**Figure 1:**
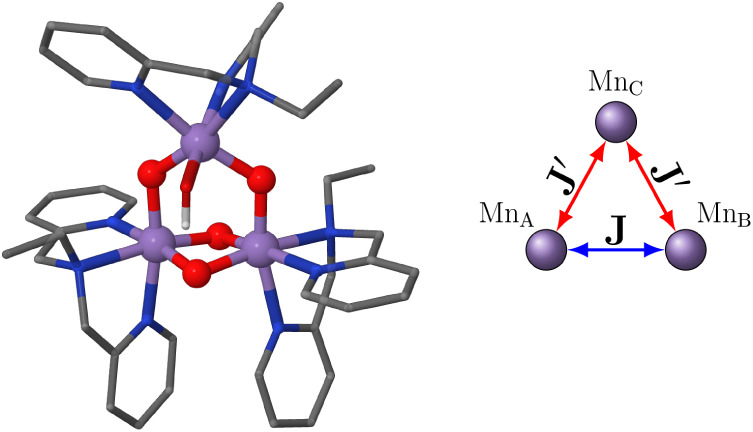
Magnetic coupling scheme in the [Mn­(IV)_3_O_4_]^3+^ cluster. Left: actual cluster with three spin 3/2 Mn­(IV) ions. Right: simplified isosceles triangle schematic highlighting the antiferromagnetic exchange constants *J* = −76 cm^–1^ (blue) and *J*′ = −11 cm^–1^ (red).^94^

Trinuclear magnetic systems have attracted considerable attention due to their unusual magnetic^97^ and biological^98^ properties, with renewed technological interest arising from the potential generation of spin-chirality qubits.^97,99^ The interplay of spin frustration and antisymmetric exchange interactions makes these architectures particularly suitable for extending the multispin picture originally proposed by Kambe.^100^ For high-spin d^3^ ions such as Mn­(IV), much of the magnetic behavior can be captured by a Heisenberg–Dirac–van Vleck spin model. For the complex considered here, an isosceles two-parameter HDvV Hamiltonian, built on local spin *S*_local_ = 3/2 for each Mn­(IV) ion, reproduces the temperature-dependent magnetic susceptibility measurements with antiferromagnetic exchange coupling constants *J* = −76 cm^–1^ and *J*′ = −11 cm^–1^ (Figure 1).^94^

Accordingly, we performed DDCI calculations based on CAS reference wave functions of increasing size to (a) validate the spin Hamiltonian used to fit the experimental temperature-dependent magnetic susceptibility, which is commonly employed to extract exchange coupling constants, and (b) assess the performance and robustness of the Stochastic-DDCI implementation.

## 2 Theory

In this section, we present the fundamental theoretical concepts that are prerequisite to the present work, namely (a) the GAS concept, which facilitates flexible partitioning of configurational spaces, and (b) the concept of DDCI and how it interfaces with GAS. The discussion of stochastic concepts has been omitted in favor of clarity and conciseness, as these aspects are covered extensively in the literature.^60,69,81,101,102^

### 2.1 Generalized Active Space

A common extension of the CAS formalism is the *restricted active space* (RAS)^103^ approach, in which orbitals are divided into three subspaces—RAS1, RAS2, and RAS3—typically corresponding to doubly occupied, partially occupied, and virtual orbitals, respectively in the reference wave function. The size of the RAS configurational space is controlled by limiting the maximum number of holes in RAS1 and particles in RAS3.^104^ The *generalized active space* (GAS)^52^ concept lifts the restriction of having either one (CAS) or three (RAS) subspaces, allowing in principle an arbitrary number *k* of subspaces, chosen on the basis of the chemical process under investigation. Active orbitals are distributed among the *k* subspaces, and for each GAS subspace, *i*, electron occupations are constrained, enabling the construction of truncated CI expansions. GAS subspaces are termed *disconnected* if no interspace excitations are allowed, and *connected* if excitations between subspaces are permitted. A single GAS wave function may contain both connected and disconnected subspaces simultaneously (see Figure 2).

**Figure 2:**
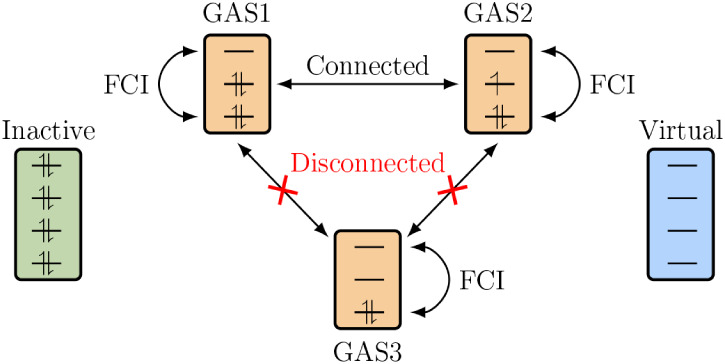
Graphical representation of a GAS space with *k* = 3 GAS subspaces. GAS1 and GAS2 are connected whereas GAS3 is disconnected.

An illustrative example of GAS is its application to X-ray absorption spectroscopy (XAS).^139^ In XAS, the target state corresponds to a core-hole excitation, where a core (K-edge) or semicore (L-edge) electron is promoted to valence levels. Within the core–valence-separated model of Cederbaum,^105^ the core-hole excited state can be naturally represented using a GAS construct. One GAS subspace (GAS1) comprises the core orbitals, while a second subspace (GAS2) contains the valence and virtual orbitals. The electron occupation of GAS1 is constrained to maintain a single hole in the core orbitals. As a result, the CI expansion includes only configurations with a core hole, ensuring that the variational optimization of the multiconfigurational wave function is selectively tailored toward the core-hole excited state. The allowed electron occupations of GAS subspaces are specified through either *local* (eq 2) or *cumulative* (eq 3) constraints:^69^

2 $$\forall i\;,1\leq i\leq k:N_{i}^{\min}\leq x_{i}\leq N_{i}^{\max}$$

3 $$\forall i,1\leq i\leq k:{Ñ}_{i}^{\min}\leq \overset{i}{\underset{j=1}{\sum }}x_{j}\leq {Ñ}_{i}^{\max}$$

Here, *x*_*i*_ denotes the occupation number of the *i*-th GAS subspace, while $\left(N_{i}^{\min},N_{i}^{\max}\right)$ and $\left({Ñ}_{i}^{\min},{Ñ}_{i}^{\max}\right)$ define the lower and upper bounds for the local and cumulative constraints, respectively. Local constraints restrict each subspace individually, whereas cumulative constraints enforce limits on the sum of occupations across multiple consecutive subspaces.

The constraints defined in eqs 2 and 3 are encoded via a list of unique sets of occupation numbers across the GAS subspaces, referred to as *supergroups*.^106,107^ In the special case where electrons are free to move among all subspaces, the set of supergroups corresponds to all integer compositions^69,108^ satisfying

4 $$x_{1}+x_{2}+...+x_{k}=N,,x_{i},N\in N_{0},k\in N$$

Under these conditions, the GAS wave function is equivalent to a CAS wave function with *N* electrons, irrespective of how the active orbitals are partitioned into subspaces. Conversely, when no interspace excitations are allowed (disconnected subspaces), only a single supergroup exists. More generally, GAS wave functions correspond to a subset of all possible compositions, with the permitted supergroups determined by the local or cumulative constraints.

Within the Stochastic-GAS framework, we have implemented an additional level of flexibility by allowing the user to directly specify the set of desired supergroups. This capability enables the construction of configurational spaces that cannot be constrained using conventional local or cumulative GAS constraints. This feature is particularly critical for DDCI calculations, where the relevant configurations are defined not by simple occupation limits but by specific classes of excitations that drive magnetic exchange (see below). By selecting the appropriate supergroups, Stochastic-GAS can faithfully reproduce the DDCI space, ensuring that the stochastic sampling targets precisely the excitations that govern the low-energy spin physics.

### 2.2 Difference-Dedicated Configuration Interaction

As detailed in the original work,^89^ DDCI arises from a second-order perturbation theory analysis. The orbital space is partitioned into three subspaces: an *inactive* space consisting of *n*_0_ doubly occupied orbitals, a partially occupied CAS­(*N*_a_,*n*_a_) reference active space, and a *virtual* space with *n*_v_ empty orbitals. Excitations beyond the CAS reference are classified according to the number of holes *h* and particles *p* generated in the inactive and virtual spaces, respectively (Figure 3).

**Figure 3:**
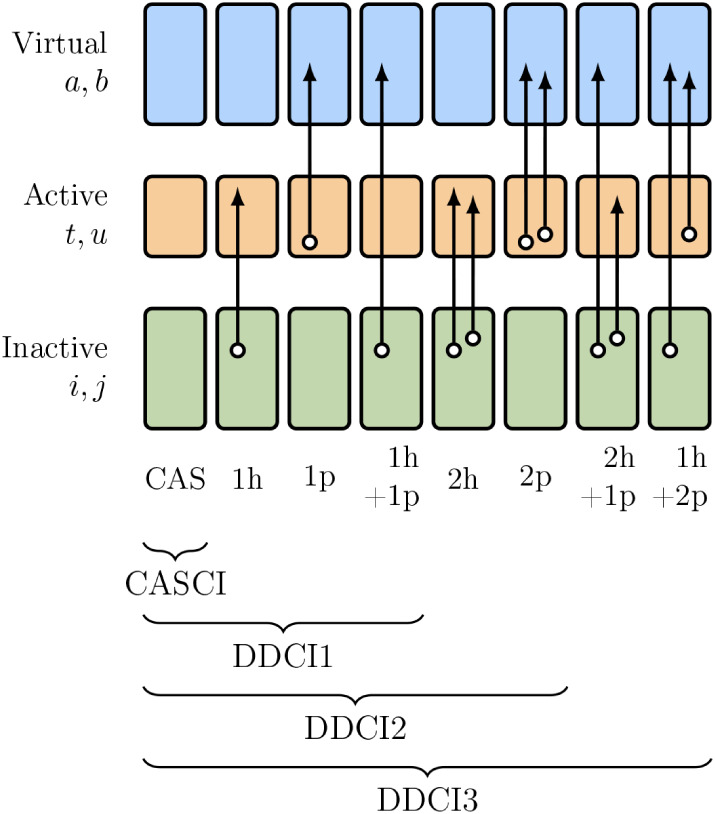
Graphical representation of the excitation classes defining the DDCI hierarchy of truncation levels.

For example, an excitation that transfers one electron from the inactive subspace into the CAS reference is classified as 1*h*, whereas an excitation from the inactive to the virtual space is classified as 1*h* + 1*p*. The DDCI many-body basis is constructed according to a hierarchy of truncation levels. DDCI1 contains 1*h*, 1*p*, and 1*h* + 1*p* excitations (also denoted CAS + S). DDCI2 additionally includes 2*h* and 2*p* excitations. DDCI3 further incorporates 2*h* + 1*p* and 1*h* + 2*p* excitations (often denoted simply as DDCI), where each higher-order level contains all configurations of the lower orders (Figure 3).

Consider the construction of an effective Hamiltonian, **H̃**, within the CAS reference space using Rayleigh–Schrödinger second-order perturbation theory.^109^ Its matrix elements are given by

5 $$\left\langle \phi_{I}\left|\mathrm{H̃}\right|\phi_{J}\right\rangle =\left\langle \phi_{I}\left|H\right|\phi_{J}\right\rangle +\underset{\alpha \in Q}{\sum }\frac{\left\langle \phi_{I}\left|H\right|\phi_{\alpha }\right\rangle \left\langle \phi_{\alpha }\left|H\right|\phi_{J}\right\rangle }{E_{J}-E_{\alpha }}$$

where the index α runs over the external configuration space Q. This space contains all configurations obtained from the CAS reference by generating up to two holes in the inactive orbitals and up to two particles in the virtual orbitals, commonly referred to as the CAS + SD space. Among these excitations, configurations obtained by promoting two electrons from inactive orbitals *i* and *j* to virtual orbitals *a* and *b* deserve particular consideration. Such determinants can be written as $\left|\phi_{\alpha }\right\rangle ={â}_{a}^{†}{â}_{b}^{†}{â}_{i}{â}_{j}\left|\phi_{I}\right\rangle$ . If |ϕ_*I*_⟩ ≠ |ϕ_*J*_⟩, the determinants |ϕ_α_⟩ and |ϕ_*J*_⟩ differ by more than two spin orbitals. Consequently, in virtue of the Slater–Condon rules, the Hamiltonian matrix element ⟨ϕ_α_|*H*|ϕ_*J*_⟩ vanishes, and these 2*h* + 2*p* configurations do not contribute to the off-diagonal elements of **H̃**. The contribution of the 2*h* + 2*p* excitations therefore reduces to diagonal energy shifts of the CAS reference configurations,

6 $$\underset{\alpha }{\sum }\frac{\left\langle \phi_{I}\left|H\right|\phi_{\alpha }\right\rangle \left\langle \phi_{\alpha }\left|H\right|\phi_{I}\right\rangle }{E_{I}-E_{\alpha }}=\underset{\begin{array}{l} i,j\in \;\mathrm{Inactive}\\ a,b\in \;\mathrm{Virtual} \end{array}}{\sum }\frac{\langle ab\left|\right|ij\rangle^{2}}{\Delta_{ab\leftarrow ij}}$$

where *E*_*I*_ – *E*_α_ = *E*_*I*_ – (*E*_*I*_ + ϵ_*a*_ + ϵ_*b*_ – ϵ_*i*_ – ϵ_*j*_) = Δ_*ab*←*ij*_ and ϵ_ν_ (ν ∈ {*a*, *b*, *i*, *j*}) denotes orbital energies. When a common set of molecular orbitals is used to describe all electronic states simultaneously, the contribution in eq 6 produces a constant shift of all CAS energies. Therefore, since these 2*h* + 2*p* excitations do not provide differential contributions to energy gaps between the target spin states, they are excluded altogether from the DDCI configurational space. It should be stressed that while DDCI is based on PT2 arguments, the method remains entirely variational.

At the computational level, the 2*h* + 2*p* excitations represent a substantial fraction of the CAS + SD configuration space. Their number scales (to the leading term) as $M\cdot n_{\mathrm{inact}}^{2}\cdot n_{\mathrm{virt}}^{2}$ , where *M* is the size of the CAS reference.^89^ Once these configurations are removed the effective scaling of the DDCI expansion is reduced to $M\cdot n_{\mathrm{inact}}\cdot n_{\mathrm{act}}\cdot n_{\mathrm{virt}}^{2}$ . This reduction is particularly important in practice since *n*_act_ ≪ *n*_inact_ in typical DDCI calculations. Eliminating these excitations is therefore generally computationally advantageous. This advantage also applies to stochastic procedures, where such configurations would otherwise be sampled through off-diagonal contributions during the *spawning* stage and diagonal contributions during the *death* stage despite providing no differential contributions.

In constructing Stochastic-DDCI, the inactive, active and virtual orbitals naturally define three GAS subspaces. The DDCI1, DDCI2 and DDCI3 truncation levels can then be encoded by the corresponding sets of supergroups. Let the set of supergroups be **x** = [*x*_0_, *x*_a_, *x*_v_] = [2*n*_0_ – *h*, *N*_a_ + (*h* – *p*), *p*]; the DDCI hierarchy is then encoded by eqs 7–9:

7 DDCI1:⁣h,p∈{0,1}

8 DDCI2:⁣h,p∈{0,1,2},(h,p)∉{(1,2),(2,1),(2,2)}

9 DDCI3:⁣h,p∈{0,1,2},⁣(h,p)∉{(2,2)}

While the conventional DDCI algorithm has been implemented and optimized for the Slater determinant (SD) many-body basis, Stochastic-DDCI has been implemented for both SD and spin-adapted Graphical Unitary Group Approach (GUGA)^62,110–113^ bases. Using a spin-adapted basis is particularly advantageous for PNTM clusters, as it allows one to directly target states with a specific total spin. Furthermore, GUGA enables the use of *Quantum Anamorphosis* ^114^ to construct a highly sparse, block-diagonal Hamiltonian^11,67,115–120^ which could be exploited to further enhance Stochastic-DDCI efficiency. Conversely, SD-based methods variationally collapse into the lowest state that can be reached by the selected *S*_*z*_, which may not correspond to the intended *S* ^2^ value. In conventional DDCI, this issue is typically circumvented by optimizing multiple roots for a given *S*_*z*_ and selecting a posteriori the one with the desired *S* ^2^ value. However, this procedure is computationally costly, as it requires optimizing redundant states corresponding to the same *S* ^2^ but different *S*_*z*_ values. Stochastic-DDCI in the SD basis provides an alternative spin-purification strategy using a first-order spin-penalty approach,^95^ which targets states with a specific *S* ^2^ value without optimizing unnecessary states, thereby further improving computational efficiency.

The implementation within the framework of Stochastic-GAS, leads to Stochastic-DDCI inheriting all features and optimizations that arise from past Stochastic-GAS developments such as the exploitation of locality effects in the excitation generator^65,121^ or compatibility with a recently developed uncontracted second order perturbation theory strategy.^81^

## 3 Applications

We present three DDCI setups to compute the *S* = {1/2,···,9/2} spin-ladder of the [Mn(IV)_3_O_4_]^3+^ cluster, demonstrating both the accuracy of Stochastic-DDCI and its ability to handle substantially larger CAS reference spaces than conventional implementations.

### 3.1 Computational Details

All steps, including orbital optimization via CASSCF, localization, and the generation of FCIDUMP files (containing the one- and two-electron integrals in the MO basis), were performed using the OpenMolcas package.^122^ Nonstochastic DDCI reference calculations were performed with the CASDI^96^ implementation in the Cost_package software.^123^ Stochastic-DDCI calculations in GUGA basis were performed using the NECI program.^101^ The Fermionic sign problem was controlled via the initiator approximation.^124^ Stochastic energies were analyzed using blocking analysis.^125^ We employ an improved Pre-Computed Heat-Bath excitation generator to efficiently sample the active space consisting of atom-localized orbitals.^65^ We adopt the nomenclature Stochastic-DDCI*X*(*a*,*b*)//(*c*,*d*), where (*a*,*b*) denote the number of electrons and orbitals in the CAS reference, (*c*,*d*) denote the total number of considered electrons and orbitals including inactive and virtual spaces, and *X* indicates the DDCI truncation level. All calculations were performed in C_1_ point-group symmetry. The Cartesian coordinates of the model system investigated have been extracted from ref 94.

#### 3.1.1 Basis Set

Manganese and oxygen atoms were described using an ANO-RCC-VDZP^126,127^ basis while the remaining atoms were only considered via an ANO-RCC-MB^126^ basis.

#### 3.1.2 Molecular Orbital Basis

Initial orbitals were obtained from a high-spin (*S* = 9/2) CASSCF­(9,9) procedure (a single-configuration ROHF-SCF), with the active space comprising the *t*_2g_ orbitals on each manganese­(IV) center and their nine electrons. A Pipek–Mezey split-localization procedure was subsequently applied, in which the inactive orbitals (the 71 core orbitals were kept frozen), the 9 active, and all virtual orbitals were localized separately, yielding an orbital transformation invariant with respect to the CASSCF­(9,9). Of the localized orbitals, 23 doubly occupied orbitals were selected as inactive, including the 12 bridging O 2p orbitals, the 9 σ orbitals of the peripheral ligands, and 2 orbitals on the apical −OH group (1 σ pointing toward the metal and 1 π orbital perpendicular to the O–H bond and lying in the basal Mn–Mn plane). The 9 *t*_2g_ magnetic orbitals and their 9 electrons were assigned to the active space. Finally, the 6 additional *e*_g_, the 15 double-shell correlating *d*′, and the 12 correlating *p*′ orbitals of the bridging atoms, were selected among the virtual space, either to build larger reference wave functions or for inclusion in the DDCI correction. Figure 4 illustrates the orbitals used to define the three reference wave functions considered in this work (details below).

**Figure 4:**
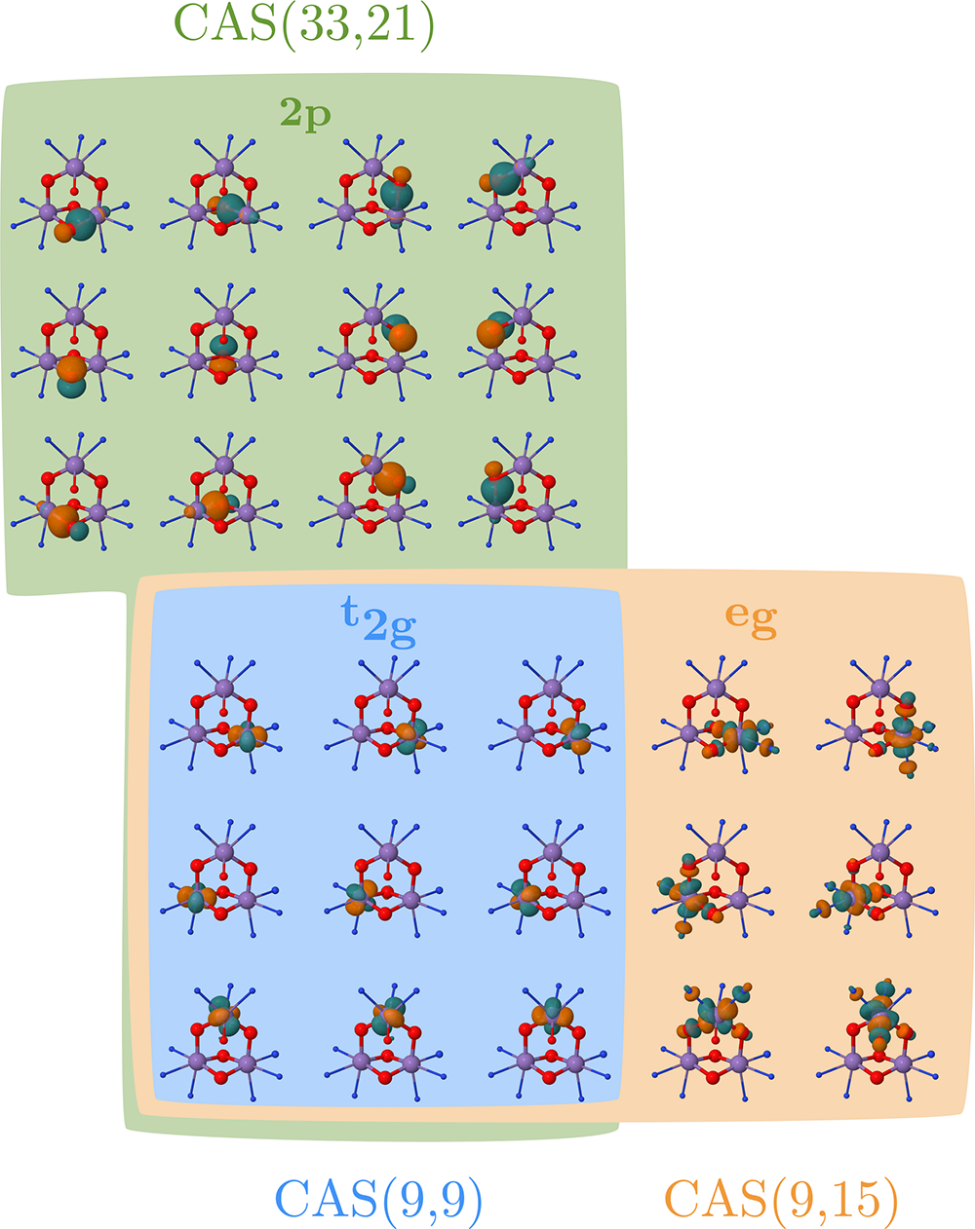
Molecular orbitals included in the CAS reference.

#### 3.1.3 CAS­(9,9) Reference Space

In the smallest model, the reference wave function is defined by a CAS­(9,9) space, comprising the 9 singly occupied *t*_2g_ magnetic orbitals and their 9 electrons. The 23 localized inactive orbitals on the bridging and peripheral oxygen atoms (46 electrons) and the 6 virtual *e*_g_ orbitals are retained as the inactive and virtual spaces, respectively, for the subsequent DDCI corrections. Consequently, a total of 55 electrons are correlated over 38 orbitals at the DDCI level, denoted as DDCI­(9,9)//(55,38) hereafter. Table 1 lists the corresponding supergroups defining the DDCI1, DDCI2, and DDCI3 expansions.

**Table 1:** List of Supergroups (See Eqs 7–9) for the DDCI­(9,9)//(55,38) and the Stochastic-DDCI­(9,15)//(55,53) Calculations

	Supergroups		
Type	DDCI1	DDCI2	DDCI3
CAS	[46,9,0]	[46,9,0]	[46,9,0]
1*h*	[45,10,0]	[45,10,0]	[45,10,0]
1*p*	[46,8,1]	[46,8,1]	[46,8,1]
1*h* + 1*p*	[45,9,1]	[45,9,1]	[45,9,1]
2*h*		[44,11,0]	[44,11,0]
2*p*		[46,7,2]	[46,7,2]
2*h* + 1*p*			[44,10,1]
1*h* + 2*p*			[45,8,2]

Considering, the relatively small expansion both in the CAS­(9,9) reference and the DDCI space, these calculations could be performed using both the nonstochastic and the Stochastic-DDCI implementations to validate the stochastic approach.

#### 3.1.4 CAS­(9,15) Reference Space

In the intermediate Stochastic-DDCI­(9,15)//(55,53) model, the reference wave function is expanded to a CAS­(9,15) by including the 6 *e*_g_ empty orbitals in the active space. The virtual space is further enlarged by adding the correlating *d*′ orbitals. The inactive space remains unchanged relative to the smaller CAS­(9,9) model. The lists of supergroups reported in Table 1 are also valid for this model space.

#### 3.1.5 CAS­(33,21) Reference Space

In the largest Stochastic-DDCI­(33,21)//(55,65) model, the reference wave function is expanded to a CAS­(33,21) by including excitations from the 12 bridging O 2p orbitals. No additional inactive orbitals are considered, leaving the remaining 11 peripheral O orbitals as the sole inactive orbitals at the DDCI level. The virtual space is further enlarged by the inclusion of the bridging *p*′ double-shell orbitals, resulting in 33 virtual orbitals. Consequently, 55 electrons are correlated over 65 orbitals in the largest Stochastic-DDCI model. The full lists of supergroups corresponding to DDCI1-DDCI3 are reported in Table 2. However, only the Stochastic-DDCI1­(33,21)//(55,65) results are reported in this work, as they already yield a spin ladder in excellent agreement with the reference (vide infra).

**Table 2:** List of Supergroups (See Eqs 7–9) for the Stochastic-DDCI­(33,21)//(55,65) Calculations

	Supergroups		
Type	DDCI1	DDCI2	DDCI3
CAS	[22,33,0]	[22,33,0]	[22,33,0]
1*h*	[21,34,0]	[21,34,0]	[21,34,0]
1*p*	[22,32,1]	[22,32,1]	[22,32,1]
1*h* + 1*p*	[21,33,1]	[21,33,1]	[21,33,1]
2*h*		[20,35,0]	[20,35,0]
2*p*		[22,31,2]	[22,31,2]
2*h* + 1*p*			[20,34,1]
1*h* + 2*p*			[21,32,2]

The largest Stochastic-DDCI­(33,21)//(55,65) space presented here results from a chemically motivated orbital selection and does not represent the upper limit of Stochastic-DDCI. For context, the largest Stochastic-GAS calculation reported to date correlated 96 electrons in 159 orbitals within an MRCI-SD framework, based on a CAS­(32,34) multireference space. Stochastic-DDCI is expected to surpass this limit, due to the systematic exclusion of the numerous 2*h*+2*p* excitations.^69,81^ Additionally, the sparse data structures inherent to the stochastic algorithm could, once fully implemented, enable the treatment of several hundred orbitals.^81^ Another promising strategy to further extend the applicability of Stochastic-DDCI is its combination with uncontracted stochastic perturbation theory in the form of Stochastic-SplitGAS.^81^

#### 3.1.6 Orbital Bias

Although using high-spin orbitals generally leads to an unbalanced description of low-spin states, favoring the high-spin solution and thereby overstabilizing the former, this effect is largely mitigated at the wave function level by employing large active space references and through the addition of the DDCI corrections. Consistently with this picture, it has been demonstrated that state-averaged orbitals have a small impact on uncontracted CI methods, including DDCI, since orbital relaxation is already effectively recovered at the CI level through the coupling between the reference wave function and its excited configurations.^128^ Another strategy to remove orbital-induced bias within the DDCI framework is *iterative*-DDCI,^129,130^ where the orbitals are iteratively optimized via the averaged density matrix across multiple states obtained from successive DDCI calculations. This strategy is fully compatible with the proposed stochastic framework, since density matrices are accessible^131^ and are routinely used in other stochastic approaches;^66,69,70^ however, its implementation lies beyond the scope of this manuscript.

#### 3.1.7 Computational Cost

No rigorous benchmarking of the computational cost of Stochastic-DDCI is provided in this work, as it greatly depends on the parametrization of the stochastic algorithm and the system under study. However, as a point of reference, the most costly *S* = 1/2 simulations representing the wave function with 5 × 10^8^ walkers running on 512 cores across 8 AMD EPYC 9554 CPU’s is able to update the wave function 25,000–30,000 times in 48 h which is generally sufficient to get an energy independent of statistical and time-correlation errors. The convergence behavior of Stochastic-DDCI*X*(9,9)/(55,38) and Stochastic-DDCI1­(33,21)/(55,65) with respect to the number of walkers is given in Figures SI.S1 and SI.S2.

### 3.2 Results and Discussion

In the following, we aim to reproduce the energetically lowest spin-ladder (*S* = 1/2 to *S* = 9/2) predicted by a 2-parameter HDvV spin Hamiltonian (*J* = −76 and *J*′ = −11 cm^–1^) extracted from experimental temperature-dependent magnetic susceptibility measurements.^94^ Details on the connection between the spin model and magnetic susceptibility can be found in Refs. 11,132 According to this spin Hamiltonian, the ground state of the system is a collinear *S*_total_ = 3/2 state, with the basal magnetic centers dominantly coupled to an overall singlet (*S*_AB_ = 0) with vanishing contributions from configurations with *S*_AB_ ∈ {1,2,3}. The *S*_total_ = 1/2 state is at about 97 cm^–1^ above the ground state, followed by the *S*_total_ = 5/2 state at 185 cm^–1^, and the *S*_total_ = 7/2 and *S*_total_ = 9/2 states at 522 cm^–1^ and 1011 cm^–1^, respectively. This ordering makes the characterization of the *S*_total_ = 1/2 state nontrivial for nonspin-adapted methods. While states with *S*_total_ ≥ 3/2 can be targeted directly by specifying the highest *M*_*S*_ value, the *S*_total_ = 1/2 state cannot be obtained simply by setting *M*_*S*_ = 1/2, which instead converges to the lower-energy *S*_total_ = 3/2 state (with *M*_*S*_ = 1/2). In conventional DDCI, this complication is handled via the multiroot optimization strategy discussed above. Spin-adapted Stochastic-DDCI, on the other hand, allows the *S*_total_ = 1/2 state to be targeted directly by specifying the appropriate total spin value.

Figure 5 summarizes the spin-ladder energetics for all methods discussed in this work, namely CASCI­(9,9), CASCI­(9,15) and Stochastic-CASCI­(33,21) as well as the corresponding Stochastic-DDCI spaces. For the small references, DDCI1, DDCI2 and DDCI3 are shown, while for the largest reference space, only the Stochastic-DDCI1­(33,21)//(55,65) is reported.

**Figure 5:**
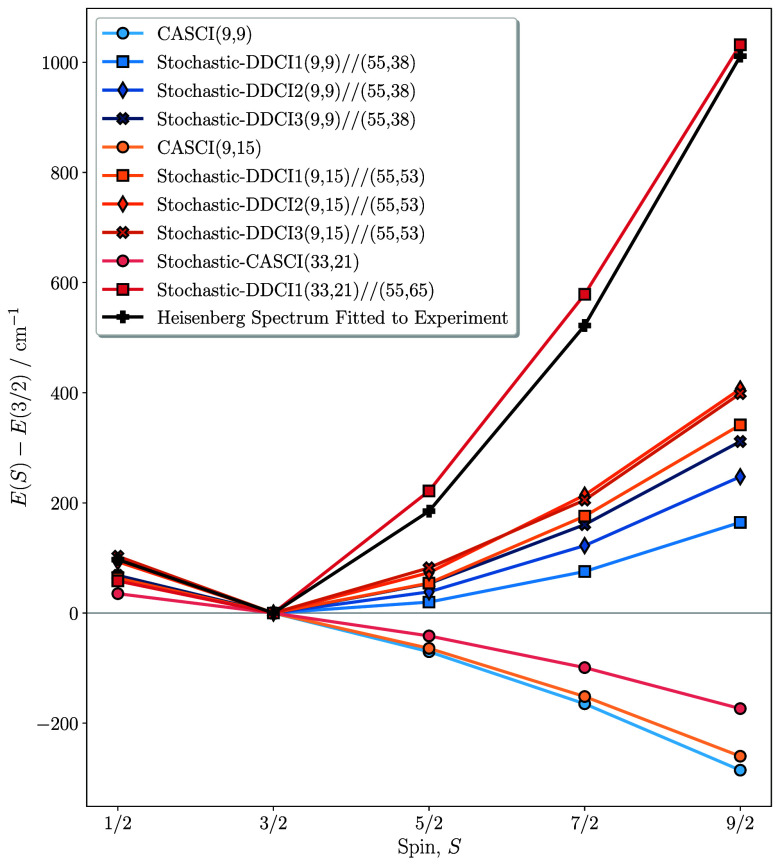
Spin ladders using various CASCI references and their respective Stochastic-DDCI schemes. The spin state *S* = 3/2 is chosen as the zero energy.

#### 3.2.1 CASCI Results

Without DDCI corrections, all CASCI simulations, independently of their size, produce qualitatively incorrect spin ladders, showing a ferromagnetic ordering of the states. The larger spaces including the Stochastic-CASCI­(33,21) marginally reduce the ferromagnetic ordering, without removing the methodological bias.

#### 3.2.2 Stochastic-DDCI

In contrast, all levels of DDCI corrections yield a spin ladder qualitatively in agreement with the reference, correctly predicting a *S* = 3/2 ground state.^94^ For Stochastic-DDCI­(9,9)//(55,38) and Stochastic-DDCI­(9,15)//(55,53), increasing the excitation level from DDCI1 to DDCI3 differentially stabilizes the low-spin states, improving agreement with the reference. However, using these small reference spaces, the DDCI correction is insufficient to fully reproduce the reference spin ladder. The lowest-to-highest spin gap are underestimated by approximately 700 cm^–1^ and 600 cm^–1^ for Stochastic-DDCI3­(9,9) and Stochastic-DDCI3­(9,15), respectively. Stochastic-DDCI results for the minimal CAS­(9,9) reference are indistinguishable from their nonstochastic counterparts (see Supporting Materials), indicating that this approach provides a scalable and reliable alternative to conventional DDCI for larger, more complex spaces that are otherwise inaccessible to conventional implementations. Due to the size of the reference space, the corresponding deterministic DDCI­(9,15) calculations were not performed, as they would require impractically large computational resources.

Stochastic-DDCI allows the reference space to be expanded well beyond the limits of the conventional DDCI implementation. In Stochastic-DDCI­(33,21)//(55,65), both the 3d *t*_2g_ magnetic and the O 2p bridging orbitals are included in the CAS­(33,21) reference space. Thus, ligand-to-metal charge-transfer configurations, which are crucial for an accurate description of the ligand-mediated superexchange mechanism, are already explicitly included in the reference wave function. For smaller CAS references, these configurations lie outside the reference space and are incorporated only through the DDCI excitations. Achieving high accuracy therefore requires sufficiently high excitation levels, ideally DDCI3, to recover the missing correlation effects. As demonstrated by the Stochastic-DDCI­(9,9) and Stochastic-DDCI­(9,15) results, however, even DDCI3 is insufficient to fully recover the required correlation, despite the inclusion of the 2*h* + 1*p* and 1*h* + 2*p* excitations. In contrast, in the larger Stochastic-DDCI­(33,21), already the DDCI1 excitation level yields a spin ladder in excellent agreement with the reference, with a root-mean-square deviation of only 33 cm^–1^ (see Appendix A.1). Most notably, the lowest-to-highest spin gap is reproduced with remarkable accuracy: we obtain 1032 cm^–1^, in very close agreement with the reference value of 1011 cm^–1^.

#### 3.2.3 Class-Partitioned CI

The flexible GAS framework allows for a class-partitioned analysis^133–136^ of the different excitation classes via the consideration of specific sets of supergroups. We take advantage of this feature to analyze the impact of the various interactions that comprise Stochastic-DDCI1­(33,21)//(55,65) to determine their significance in achieving the proper energetic ordering relative to the CASCI­(33,21) reference.

Figure 6 illustrates the individual impacts of 1*h*, 1*h*1*p*, and 1*p* excitations on the CAS reference. The 1*h* and 1*h*1*p* excitation classes exert a negligible influence on the energy, which is in agreement with our expectations, since they account for the interactions between the peripheral σ orbitals and the 2p and *t*_2g_ or p′, d′ and *e*_g_ orbitals, respectively. Instead, the majority of the DDCI1 correction arises from 1*p* excitations, with an average deviation of 60 cm^–1^ compared to the deviation of 33 cm^–1^ in DDCI1. This is readily explained by the choice of CAS reference. Since the CAS­(33,21) reference space includes the bridging O 2p orbitals together with the Mn *t*_2g_ shell, the corresponding Ligand-to-Metal Charge-Transfer (LMCT) configurations are already described variationally within the CASCI wave function. In particular, the active-space treatment accounts not only for single *t*_2g_ ← 2p LMCT excitations, but also for higher-order multiple CT configurations, which can contribute significantly to the low-energy spectrum.^137^ However, the virtual *e*_g_, double-shell d′, and ligand p′ orbitals are not included in the active space, such that the CASCI description lacks the orbital relaxation mechanisms associated with these external degrees of freedom. The CASCI + 1*p* treatment remedies this limitation by allowing excitations from the CAS into the virtual space, thereby introducing couplings with configurations involving d′ ← 2p, *e*_g_ ← 2p, and p′ ← 2p excitations (or the respective ← *t*_2g_ equivalents). These additional configurations do not primarily generate new LMCT effects, which are already contained in the CAS reference, but rather provide the orbital relaxation (breathing) response of the valence shell to the different charge occupations encountered in the neutral, ionic and CT configurations. In particular, the p′ ← 2p excitations effectively describe orbital rotations within the ligand space, allowing the bridging 2p orbitals to expand or contract depending on their instantaneous occupation. This has already been discussed in the literature.^138^ From a CI perspective, this also introduces higher-order configurations, such as 3*h*1*p* terms arising from orbital relaxation on top of double LMCT configurations, which are absent in the smaller CI expansions based on CAS­(9,9) and CAS­(9,15) references. However, this results indicate that the calculations based on smaller references would benefit from the inclusion of p′ and d′ in the virtual space to allow for the description of a single LMCT including orbital relaxation via 2*h*1*p* interactions at the DDCI3 level.

**Figure 6:**
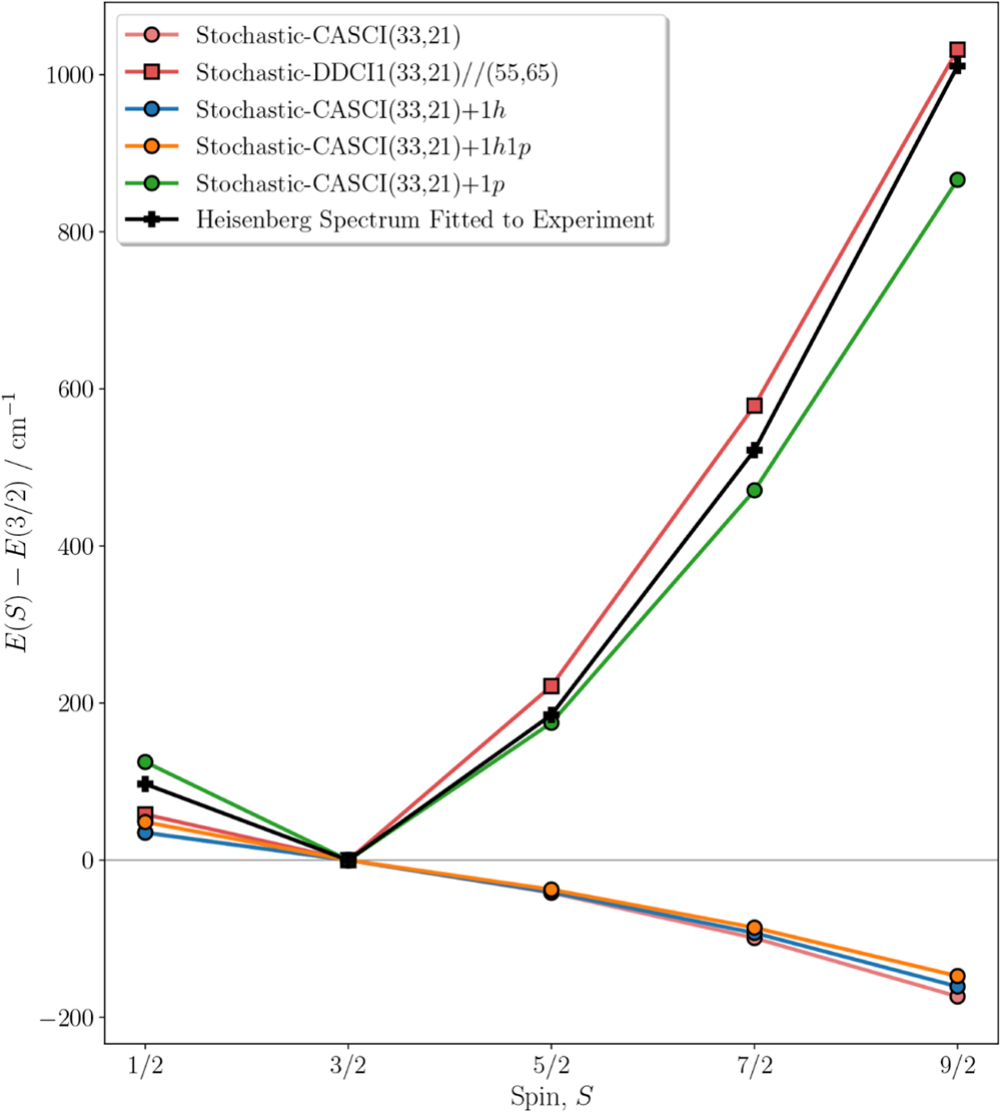
Effect of individual Stochastic-DDCI1 components on the Stochastic-CASCI­(33,21) reference wave function.

## 4 Conclusions

In this work, we presented the novel Stochastic-DDCI method, which relies on the flexible GAS constraints for excluding (2*h* + 2*p*) excitations, and the Full Configuration Interaction Quantum Monte Carlo algorithm for the stochastic optimization of the resulting truncated many-body wave function. Stochastic-DDCI addresses a different approximation problem from large-active-space approximate eigensolvers such as CAS-DMRG or CAS-FCIQMC. The latter aim at approaching the full-CI limit within a chosen active space, whereas DDCI is a physically motivated truncated CI framework designed to retain the dominant configurations contributing to magnetic exchange interactions and their associated differential dynamic-correlation effect. In principle, when computationally feasible, a converged DMRG or FCIQMC treatment of a sufficiently large active space provides a more complete description of electron correlation within that space. However, for large polynuclear transition-metal clusters, the active spaces required to simultaneously capture static and differential dynamic correlation may become prohibitively large, making fully converged calculations challenging in practice due, e.g., to the bond-dimension requirements in DMRG or the walker population needed in FCIQMC. The main advantage of Stochastic-DDCI is therefore not higher formal accuracy within a fixed active space, but rather its ability to treat very large orbital spaces while retaining the physically most relevant configurations for magnetic couplings. By exploiting the reduced dimensionality of the DDCI excitation manifold together with stochastic sampling, the method can access correlation regimes that would be difficult to converge with more complete active-space treatments. From this perspective, Stochastic-DDCI is conceptually closer to selected or perturbative approaches targeting the dominant contributions to magnetic exchange than to full active-space solvers. A more direct comparison can therefore be made with Stochastic-GAS^69^ or Stochastic-SplitGAS,^81^ where the excitation space is likewise restricted according to physically motivated criteria.

The scalability and robustness of the method has been demonstrated by applying it to the challenging [Mn­(IV)_3_O_4_]^3+^ cluster, which is a prototypical example for many-unpaired-electron magnetic systems. There, the CAS reference space was enlarged from the small CAS­(9,9), which is still accessible by standard DDCI implementations, to the CAS­(9,15) and CAS­(33,21), which are beyond the limits of standard DDCI implementations. Importantly, the largest Stochastic-DDCI­(33,21)//(55,65) model, produces a spin ladder that is in excellent agreement with the model employed to fit the experimental data, substantially improving over the smaller CAS­(9,9) and CAS­(9,15) reference spaces. This work establishes Stochastic-DDCI as a viable new Ansatz for magnetic systems with many unpaired electrons, pushing the limits toward larger and more complex structures, while retaining the conceptual simplicity of the well-established DDCI paradigm.

Particularly promising is the envisioned combination of Stochastic-DDCI with an algorithm for a sparse representation of the probability tables in the stochastic framework and the stochastic perturbation theory, both of which are expected to allow the treatment of several hundred orbitals.

## Supplementary Material





## Appendix A

### Deviation to Reference Data

The deviation of the Stochastic-DDCI spectrum to the reference data across all spins *S* ∈ {1/2, 3/2, 5/2, 7/2, 9/2} is obtained as follows. In order to compare the simulated and reference ladder, one spin state *S* is chosen as the zero-energy. However, this introduces a bias since both respective data sets are shifted by different constant values. In order to remove this bias we first calculate the mean difference with respect to the reference values, Δ_mean_,

10
$$\Delta_{\mathrm{mean}}=\frac{1}{5}\underset{i\in S}{\sum }\left(E\left(i\right)-E_{\mathrm{ref}}\left(i\right)\right)$$

Δ_mean_ is then subtracted when calculating the Root-Mean-Square Deviation Δ_RMSD_ to ensure an error estimate independent from the original zero-energy choice,

11
$$\Delta_{\mathrm{RMSD}}=\sqrt{\frac{1}{5}\underset{i\in S}{\sum }{\left(E\left(i\right)-E_{\mathrm{ref}}\left(i\right)-\Delta_{\mathrm{mean}}\right)}^{2}}$$

## Notes

All energies obtained in the discussed calculations, the employed orbitals, and the Cartesian coordinates (PDF)
Cartesian coordinates (ZIP)

## #

European Centre for Medium Range Weather Forecast, Robert Schuman Platz 3, 53175, Bonn

## References

[1] Sanakis Y., Macedo A. L., Moura I., Moura J. J., Papaefthymiou V., Münck E. (2000). Evidence for Antisymmetric Exchange in Cuboidal [3Fe-4S]^+^ Clusters. J. Am. Chem. Soc..

[2] Shi K., Mathivathanan L., Boudalis A. K., Turek P., Chakraborty I., Raptis R. G. (2019). Nitrite reduction by trinuclear copper pyrazolate complexes:
An example of a catalytic, synthetic polynuclear NO releasing system. Inorg. Chem..

[3] Seo J. S., Whang D., Lee H., Jun S. I., Oh J., Jeon Y. J., Kim K. (2000). A homochiral metal–organic
porous material for enantioselective separation and catalysis. Nature.

[4] Armentano D., Mastropietro T. F., De Munno G., Rossi P., Lloret F., Julve M. (2008). New extended magnetic systems based on oxalate and iron (III) ions. Inorg. Chem..

[5] Sessoli R., Tsai H. L., Schake A. R., Wang S., Vincent J. B., Folting K., Gatteschi D., Christou G., Hendrickson D. N. (1993). High-spin
molecules: [Mn_12_O_12_(O_2_CR)_16_(H_2_O)_4_]. J. Am. Chem.
Soc..

[6] Chiesa A., Guidi T., Carretta S., Ansbro S., Timco G. A., Vitorica-Yrezabal I., Garlatti E., Amoretti G., Winpenny R. E. P., Santini P. (2017). Magnetic Exchange
Interactions in the Molecular Nanomagnet
Mn_12_. Phys. Rev. Lett..

[7] Mavragani N., Murugesu M. (2025). Mn_12_ and
the dawn of single-molecule magnets. Nat. Chem..

[8] Coronado E., Galan-Mascaros J. R., Gómez-García C. J., Laukhin V. (2000). Coexistence
of ferromagnetism and metallic conductivity in a molecule-based layered
compound. Nature.

[9] Williams J. M., Schultz A. J., Geiser U., Carlson K. D., Kini A. M., Wang H. G., Kwok W.-K., Whangbo M.-H., Schirber J. E. (1991). Organic
superconductors–New benchmarks. Science.

[10] Krzton-Maziopa A. (2021). Intercalated
iron chalcogenides: phase separation phenomena and superconducting
properties. Front. Chem..

[11] Li
Manni G. (2021). Modeling magnetic interactions in high-valent trinuclear complexes
through highly compressed multi-configurational wave functions. Phys. Chem. Chem. Phys..

[12] Cremades E., Ruiz E. (2010). Magnetic Properties of Largest-Spin Single Molecule Magnets: Mn_17_ Complexes – A Density Functional Theory Approach. Inorg. Chem..

[13] Caballol R., Castell O., Illas F., de P. R. Moreira I., Malrieu J. P. (1997). Remarks on the proper use of the broken symmetry approach
to magnetic coupling. J. Phys. Chem. A.

[14] Calzado C. J., Malrieu J.-P. (2004). Origin and evaluation
of the four-spin operators in
magnetic lattices. Phys. Rev. B.

[15] Moreira I. D. P., Calzado C. J., Malrieu J.-P., Illas F. (2006). First-Principles Periodic
Calculation of Four-Body Spin Terms in High-*T_c_
* Cuprate Superconductors. Phys. Rev. Lett..

[16] Abbati G. L., Brunel L.-C., Casalta H., Cornia A., Fabretti A. C., Gatteschi D., Hassan A. K., Jansen A. G., Maniero A. L., Pardi L. (2001). Single-Ion versus Dipolar Origin of the Magnetic Anisotropy
in Iron (III)-Oxo Clusters: A Case Study. Chem.
Eur. J..

[17] Van
Slageren J., Sessoli R., Gatteschi D., Smith A. A., Helliwell M., Winpenny R. E., Cornia A., Barra A.-L., Jansen A. G., Rentschler E. (2002). Magnetic anisotropy of the antiferromagnetic ring [Cr_8_F_8_Piv_16_]. Chem. Eur.
J..

[18] White S. R. (1992). Density
matrix formulation for quantum renormalization groups. Phys. Rev. Lett..

[19] White S. R. (1993). Density-matrix
algorithms for quantum renormalization groups. Phys. Rev. B.

[20] White S. R., Martin R. L. (1999). Ab initio quantum chemistry using the density matrix
renormalization group. J. Chem. Phys..

[21] Chan G. K.-L., Head-Gordon M. (2002). Highly correlated
calculations with a polynomial cost
algorithm: A study of the density matrix renormalization group. J. Chem. Phys..

[22] Chan G. K.-L. (2004). An algorithm
for large scale density matrix renormalization group calculations. J. Chem. Phys..

[23] Zgid D., Nooijen M. (2008). The density matrix renormalization group self-consistent
field method: Orbital optimization with the density matrix renormalization
group method in the active space. J. Chem. Phys..

[24] Ghosh D., Hachmann J., Yanai T., Chan G. K.-L. (2008). Orbital optimization
in the density matrix renormalization group, with applications to
polyenes and *β*-carotene. J. Chem. Phys..

[25] Marti K. H., Reiher M. (2010). The Density Matrix Renormalization Group Algorithm
in Quantum Chemistry. Z. Phys. Chem..

[26] Schollwöck U. (2011). The density-matrix
renormalization group in the age of matrix product states. Ann. Phys..

[27] Chan G. K.-L., Sharma S. (2011). The Density Matrix Renormalization
Group in Quantum
Chemistry. Annu. Rev. Phys. Chem..

[28] Sharma S., Chan G. K.-L. (2012). Spin-adapted
density matrix renormalization group algorithms
for quantum chemistry. J. Chem. Phys..

[29] Keller S., Dolfi M., Troyer M., Reiher M. (2015). An efficient matrix
product operator representation of the quantum chemical Hamiltonian. J. Chem. Phys..

[30] Ma Y., Knecht S., Keller S., Reiher M. (2017). Second-Order Self-Consistent-Field
Density-Matrix Renormalization Group. J. Chem.
Theory Comput..

[31] Nakatani N., Guo S. (2017). Density matrix renormalization group
(DMRG) method as a common tool
for large active-space CASSCF/CASPT2 calculations. J. Chem. Phys..

[32] Baiardi A., Reiher M. (2020). The density matrix
renormalization group in chemistry
and molecular physics: Recent developments and new challenges. J. Chem. Phys..

[33] Menczer A., van Damme M., Rask A., Huntington L., Hammond J., Xantheas S. S., Ganahl M., Legeza Ö. (2024). Parallel
Implementation of the Density Matrix Renormalization Group Method
Achieving a Quarter petaFLOPS Performance on a Single DGX-H100 GPU
Node. J. Chem. Theory Comput..

[34] Brabec J., Brandejs J., Kowalski K., Xantheas S., Legeza Ö., Veis L. (2021). Massively parallel
quantum chemical density matrix
renormalization group method. J. Comput. Chem..

[35] Legeza O., Sólyom J. (2003). Optimizing
the density-matrix renormalization group
method using quantum information entropy. Phys.
Rev. B.

[36] Huron B., Malrieu J. P., Rancurel P. (1973). Iterative
perturbation calculations
of ground and excited state energies from multiconfigurational zeroth-order
wavefunctions. J. Chem. Phys..

[37] Evangelisti S., Daudey J.-P., Malrieu J.-P. (1983). Convergence
of an improved CIPSI
algorithm. Chem. Phys..

[38] Holmes A. A., Tubman N. M., Umrigar C. J. (2016). Heat-Bath
Configuration Interaction:
An Efficient Selected Configuration Interaction Algorithm Inspired
by Heat-Bath Sampling. J. Chem. Theory Comput..

[39] Tubman N. M., Lee J., Takeshita T. Y., Head-Gordon M., Whaley K. B. (2016). A deterministic
alternative to the full configuration interaction quantum Monte Carlo
method. J. Chem. Phys..

[40] Garniron Y., Scemama A., Loos P.-F., Caffarel M. (2017). Hybrid stochastic-deterministic
calculation of the second-order perturbative contribution of multireference
perturbation theory. J. Chem. Phys..

[41] Smith J. E. T., Mussard B., Holmes A. A., Sharma S. (2017). Cheap and Near Exact
CASSCF with Large Active Spaces. J. Chem. Theory
Comput..

[42] Sharma S., Holmes A. A., Jeanmairet G., Alavi A., Umrigar C. J. (2017). Semistochastic
Heat-Bath Configuration Interaction Method: Selected Configuration
Interaction with Semistochastic Perturbation Theory. J. Chem. Theory Comput..

[43] Garniron Y., Scemama A., Giner E., Caffarel M., Loos P.-F. (2018). Selected
configuration interaction dressed by perturbation. J. Chem. Phys..

[44] Levine D.
S., Hait D., Tubman N. M., Lehtola S., Whaley K. B., Head-Gordon M. (2020). CASSCF with
Extremely Large Active Spaces Using the
Adaptive Sampling Configuration Interaction Method. J. Chem. Theory Comput..

[45] Tubman N. M., Freeman C. D., Levine D. S., Hait D., Head-Gordon M., Whaley K. B. (2020). Modern Approaches to Exact Diagonalization
and Selected
Configuration Interaction with the Adaptive Sampling CI Method. J. Chem. Theory Comput..

[46] Zhang N., Liu W., Hoffmann M. R. (2020). Iterative Configuration
Interaction with Selection. J. Chem. Theory
Comput..

[47] Chilkuri V. G., Neese F. (2021). Comparison of many-particle representations for selected-CI I: A
tree based approach. J. Comput. Chem..

[48] Chilkuri V. G., Neese F. (2021). Comparison of Many-Particle
Representations for Selected Configuration
Interaction: II. Numerical Benchmark Calculations. J. Chem. Theory Comput..

[49] Evangelista F. A. (2014). Adaptive
multiconfigurational wave functions. J. Chem.
Phys..

[50] Yao Y., Umrigar C. J. (2021). Orbital Optimization
in Selected Configuration Interaction
Methods. J. Chem. Theory Comput..

[51] Park J. W. (2021). Second-Order
Orbital Optimization with Large Active Spaces Using Adaptive Sampling
Configuration Interaction (ASCI) and Its Application to Molecular
Geometry Optimization. J. Chem. Theory Comput..

[52] Ma D., Li Manni G., Gagliardi L. (2011). The generalized
active space concept
in multiconfigurational self-consistent field methods. J. Chem. Phys..

[53] Hermes M.
R., Gagliardi L. (2019). Multiconfigurational
Self-Consistent Field Theory with
Density Matrix Embedding: The Localized Active Space Self-Consistent
Field Method. J. Chem. Theory Comput..

[54] Pandharkar R., Hermes M. R., Cramer C. J., Gagliardi L. (2019). Spin-State
Ordering in Metal-Based Compounds Using the Localized Active Space
Self-Consistent Field Method. J. Phys. Chem.
Lett..

[55] Hermes M. R., Pandharkar R., Gagliardi L. (2020). Variational Localized Active Space
Self-Consistent Field Method. J. Chem. Theory
Comput..

[56] Pandharkar R., Hermes M. R., Cramer C. J., Truhlar D. G., Gagliardi L. (2021). Localized
Active Space Pair-Density Functional Theory. J. Chem. Theory Comput..

[57] Pandharkar R., Hermes M. R., Cramer C. J., Gagliardi L. (2022). Localized
Active Space-State Interaction: A Multireference Method for Chemical
Insight. J. Chem. Theory Comput..

[58] Agarawal V., King D. S., Hermes M. R., Gagliardi L. (2024). Automatic
State Interaction with Large Localized Active Spaces for Multimetallic
Systems. J. Chem. Theory Comput..

[59] Vitale E., Li Manni G., Alavi A., Kats D. (2022). FCIQMC-Tailored Distinguishable
Cluster Approach: Open-Shell Systems. J. Chem.
Theory Comput..

[60] Booth G. H., Thom A. J. W., Alavi A. (2009). Fermion Monte Carlo
without fixed
nodes: A game of life, death, and annihilation in Slater determinant
space. J. Chem. Phys..

[61] Booth G. H., Smart S. D., Alavi A. (2014). Linear-scaling
and parallelisable
algorithms for stochastic quantum chemistry. Mol. Phys..

[62] Dobrautz W., Smart S. D., Alavi A. (2019). Efficient formulation of full configuration
interaction quantum Monte Carlo in a spin eigenbasis via the graphical
unitary group approach. J. Chem. Phys..

[63] Blunt N. S., Smart S. D., Kersten J. A. F., Spencer J. S., Booth G. H., Alavi A. (2015). Semi-stochastic full
configuration interaction quantum Monte Carlo:
Developments and application. J. Chem. Phys..

[64] Blunt N. S., Smart S. D., Booth G. H., Alavi A. (2015). An excited-state approach
within full configuration interaction quantum Monte Carlo. J. Chem. Phys..

[65] Weser O., Alavi A., Li Manni G. (2023). Exploiting
Locality in Full Configuration
Interaction Quantum Monte Carlo for Fast Excitation Generation. J. Chem. Theory Comput..

[66] Li
Manni G., Smart S. D., Alavi A. (2016). Combining the Complete
Active Space Self-Consistent Field Method and the Full Configuration
Interaction Quantum Monte Carlo within a Super-CI Framework, with
Application to Challenging Metal-Porphyrins. J. Chem. Theory Comput..

[67] Dobrautz W., Weser O., Bogdanov N. A., Alavi A., Li Manni G. (2021). Spin-Pure
Stochastic-CASSCF via GUGA-FCIQMC Applied to Iron-Sulfur Clusters. J. Chem. Theory Comput..

[68] Weser O., Freitag L., Guther K., Alavi A., Li Manni G. (2021). Chemical insights
into the electronic structure of Fe­(II) porphyrin using FCIQMC, DMRG,
and generalized active spaces. Int. J. Quantum
Chem..

[69] Weser O., Guther K., Ghanem K., Li Manni G. (2022). Stochastic Generalized
Active Space Self-Consistent Field: Theory and Application. J. Chem. Theory Comput..

[70] Song M., Bonfirraro L., Galván I. F., Lindh R., Li Manni G. (2026). Spin-Adapted
Restricted Open-Shell Hartree-Fock and Its Dynamic Correlation Extension. J. Chem. Theory Comput..

[71] Safari A. A., Anderson R. J., Li Manni G. (2024). Toward a Stochastic
Complete Active
Space Second-Order Perturbation Theory. J. Phys.
Chem. A.

[72] Safari A. A., Anderson R. J., Alavi A., Li Manni G. (2025). FCIQMC-CASPT2 with
Imaginary-Time-Averaged Wave Functions. J. Chem.
Theory Comput..

[73] Anderson R. J., Shiozaki T., Booth G. H. (2020). Efficient and stochastic
multireference
perturbation theory for large active spaces within a full configuration
interaction quantum Monte Carlo framework. J.
Chem. Phys..

[74] Halson J. J., Anderson R. J., Booth G. H. (2020). Improved
stochastic multireference
perturbation theory for correlated systems with large active spaces. Mol. Phys..

[75] Kurashige Y., Yanai T. (2011). Second-order perturbation
theory with a density matrix renormalization
group self-consistent field reference function: Theory and application
to the study of chromium dimer. J. Chem. Phys..

[76] Kurashige Y. (2014). Multireference
electron correlation methods with density matrix renormalisation group
reference functions. Mol. Phys..

[77] Kurashige Y., Chalupský J., Lan T. N., Yanai T. (2014). Complete active space
second-order perturbation theory with cumulant approximation for extended
active-space wavefunction from density matrix renormalization group. J. Chem. Phys..

[78] Phung Q. M., Wouters S., Pierloot K. (2016). Cumulant Approximated
Second-Order
Perturbation Theory Based on the Density Matrix Renormalization Group
for Transition Metal Complexes: A Benchmark Study. J. Chem. Theory Comput..

[79] Phung Q. M., Pierloot K. (2019). Low-Lying Electromeric States in
Chloro-Ligated Iron­(IV)-Oxo
Porphyrin as a Model for Compound I, Studied with Second-Order Perturbation
Theory Based on Density Matrix Renormalization Group. J. Chem. Theory Comput..

[80] Phung Q. M., Muchammad Y., Yanai T., Ghosh A. (2021). A DMRG/CASPT2
Investigation
of Metallocorroles: Quantifying Ligand Noninnocence in Archetypal
3d and 4d Element Derivatives. JACS Au.

[81] Bonfirraro L., Weser O., Song M., Li Manni G. (2025). Stochastic-SplitGAS:
A Quantum Monte Carlo Multi-Reference Perturbation Theory Based on
the Imaginary-Time Evolution of Effective Hamiltonians. J. Chem. Theory Comput..

[82] Evangelista F. A. (2014). A driven
similarity renormalization group approach to quantum many-body problems. J. Chem. Phys..

[83] Li C., Evangelista F. A. (2019). Multireference
Theories of Electron Correlation Based
on the Driven Similarity Renormalization Group. Annu. Rev. Phys. Chem..

[84] Li C., Evangelista F. A. (2015). Multireference
Driven Similarity Renormalization Group:
A Second-Order Perturbative Analysis. J. Chem.
Theory Comput..

[85] Li C., Verma P., Hannon K. P., Evangelista F. A. (2017). A low-cost
approach to electronic excitation energies based on the driven similarity
renormalization group. J. Chem. Phys..

[86] Hannon K. P., Li C., Evangelista F. A. (2016). An integral-factorized
implementation
of the driven similarity renormalization group second-order multireference
perturbation theory. J. Chem. Phys..

[87] Li
Manni G., Carlson R. K., Luo S., Ma D., Olsen J., Truhlar D. G., Gagliardi L. (2014). Multiconfiguration
Pair-Density Functional Theory. J. Chem. Theory
Comput..

[88] Gagliardi L., Truhlar D. G., Li Manni G., Carlson R. K., Hoyer C. E., Bao J. L. (2017). Multiconfiguration
Pair-Density Functional Theory:
A New Way To Treat Strongly Correlated Systems. Acc. Chem. Res..

[89] Miralles J., Castell O., Caballol R., Malrieu J.-P. (1993). Specific
CI calculation
of energy differences: Transition energies and bond energies. Chem. Phys..

[90] Malrieu J. P., Caballol R., Calzado C. J., De Graaf C., Guihery N. (2014). Magnetic interactions
in molecules and highly correlated materials: physical content, analytical
derivation, and rigorous extraction of magnetic Hamiltonians. Chem. Rev..

[91] Castell O., Caballol R., García V. M., Handrick K. (1996). Ab Initio CI Determination
of the Exchange Coupling Constant of Doubly-Bridged Nickel­(II) Dimers. Inorg. Chem..

[92] Calzado C. J., Evangelisti S. (2014). Exchange interactions in [2 × 2] Cu­(II) grids:
on the reliability of the fitting spin models. Dalton Trans..

[93] Calzado C. J., Ben Amor N., Maynau D. (2014). Magnetic Coupling Constants of Self-Assembled
CuII [3 × 3] Grids: Alternative Spin Model from Theoretical Calculations. Chem. - Eur. J..

[94] Pal S., Chan M. K., Armstrong W. H. (1992). Ground
spin state variability in
manganese oxo aggregates. Demonstration of an *S* =
3/2 ground state for [Mn_3_O_4_(OH)­(bpea)_3_]­(ClO_4_)_3_. J. Am. Chem.
Soc..

[95] Weser O., Liebermann N., Kats D., Alavi A., Li Manni G. (2022). Spin Purification
in Full-CI Quantum Monte Carlo via a First-Order Penalty Approach. J. Phys. Chem. A.

[96] Ben
Amor N., Maynau D. (1998). Size-consistent self-consistent configuration interaction
from a complete active space. Chem. Phys. Lett..

[97] Trif M., Troiani F., Stepanenko D., Loss D. (2008). Spin-electric coupling
in molecular magnets. Phys. Rev. Lett..

[98] Le
Guennic B., Petit S., Chastanet G., Pilet G., Luneau D., Ben Amor N., Robert V. (2008). Antiferromagnetic
behavior based on quasi-orthogonal MOs: synthesis and characterization
of a Cu_3_ oxidase model. Inorg. Chem..

[99] Le
Mardelé F., Mohelský I., Wyzula J., Orlita M., Turek P., Troiani F., Boudalis A. K. (2025). Probing spin-electric
transitions in a molecular exchange qubit. Nat.
Commun..

[100] Kambe K. (1950). On the paramagnetic
susceptibilities of some polynuclear complex
salts. J. Phys. Soc. Jpn..

[101] Guther K., Anderson R. J., Blunt N. S., Bogdanov N. A., Cleland D., Dattani N., Dobrautz W., Ghanem K., Jeszenszki P., Liebermann N. (2020). NECI: N-Electron Configuration
Interaction with an emphasis on state-of-the-art stochastic methods. J. Chem. Phys..

[102] Li Manni, G. ; Guther, K. ; Ma, D. ; Dobrautz, W. Quantum Chemistry and Dynamics of Excited States; John Wiley & Sons, Ltd, 2020, pp. 133–203.

[103] Olsen J., Roos B. O., Jørgensen P., Jensen H. J. A. (1988). Determinant based configuration interaction algorithms
for complete and restricted configuration interaction spaces. J. Chem. Phys..

[104] Aquilante F., Autschbach J., Carlson R. K., Chibotaru L. F., Delcey M. G., De Vico L., Galván I. F., Ferré N., Frutos L. M., Gagliardi L. (2016). Molcas 8: New capabilities for multiconfigurational quantum chemical
calculations across the periodic table. J. Comput.
Chem..

[139] Alías-Rodríguez M., Montorsi F., Park W. (2026). Benchmarking Core-Level
X-ray Absorption with MRSF-TDDFT, RASPT2,
and Stochastic GAS Using the XABOOM Set. ChemRxiv.

[105] Cederbaum L. S., Domcke W., Schirmer J. (1980). Many-Body
Theory of
Core Holes. Phys. Rev. A.

[106] Olsen, J. LUCIA – A Configuration Interaction and Coupled Cluster Program; University of Århus.

[107] Li Manni, G. New Methods to Treat Strongly Correlated Systems; Ph.D. thesis, University of Geneva, 2013.

[108] Opdyke J. D. J. (2010). A Unified Approach to Algorithms Generating Unrestricted
and Restricted Integer Compositions and Integer Partitions. J. Math. Modell. Algorithms.

[109] Lindgren, I. ; Morrison, J. Atomic Many-Body Theory; Springer Science & Business Media, 2012; Vol. 3.

[110] Shavitt I. (1977). Graph theoretical
concepts for the unitary group approach
to the many-electron correlation problem. Int.
J. Quantum Chem..

[111] Shavitt I. (1978). Matrix element evaluation in the unitary group approach
to the electron correlation problem. Int. J.
Quantum Chem..

[112] Paldus, J. The Unitary Group for the Evaluation of Electronic Energy Matrix Elements; Hinze, J. Eds.; Springer: Berlin Heidelberg, 1981, pp. 1–50.

[113] Weser, O. ; Dobrautz, W. ; Li Manni, G. , (Unpublished work).

[114] Li Manni, G. QUANTUM ANAMORPHOSIS: A Quantum CheEmical Strategy Towards the Rationalization and Prediction of Magnetic Interactions in Polynuclear Transition Metal Clusters; Habilitation thesis, University of Stuttgart, 2024. https://rds-stg.ibs-bw.de/link?kid=1899104100.

[115] Li Manni G., Dobrautz W., Alavi A. (2020). Compression
of Spin-Adapted
Multiconfigurational Wave Functions in Exchange-Coupled Polynuclear
Spin Systems. J. Chem. Theory Comput..

[116] Li Manni G., Dobrautz W., Bogdanov N. A., Guther K., Alavi A. (2021). Resolution of Low-Energy States in
Spin-Exchange Transition-Metal
Clusters: Case Study of Singlet States in [Fe(III)_4_S_4_]Cubanes. J. Phys. Chem. A.

[117] Song M., Alavi A., Li Manni G. (2024). Permutation
symmetry
in spin-adapted many-body wave functions. Faraday
Discuss..

[118] Han R., Luber S., Li Manni G. (2023). Magnetic Interactions in a [Co­(II)_3_Er­(III)­(OR)_4_] Model Cubane through Forefront Multiconfigurational
Methods. J. Chem. Theory Comput..

[119] Li Manni G., Kats D., Liebermann N. (2023). Resolution
of Electronic States in Heisenberg Cluster Models within the Unitary
Group Approach. J. Chem. Theory Comput..

[120] Song M., Li Manni G. (2025). A Genetic Algorithm
Approach for
Compact Wave Function Representations in Spin-Adapted Bases. J. Chem. Theory Comput..

[121] Walker A. J. (1977). An Efficient Method for Generating Discrete Random
Variables with General Distributions. ACM Trans.
Math. Softw..

[122] Li Manni G., Galván I. F., Alavi A., Aleotti F., Aquilante F., Autschbach J., Avagliano D., Baiardi A., Bao J. J., Battaglia S. (2023). The OpenMolcas Web: A Community-Driven Approach
to Advancing Computational
Chemistry. J. Chem. Theory Comput..

[123] Ben Amor, N. ; Maynau, D. ; Pitarch-Ruiz, J.-V. ; Monari, A. ; Sophie, H. Cost_package; GitHub, http://github.com/LCPQ/Cost_package.

[124] Cleland D., Booth G. H., Alavi A. (2010). Communications:
Survival
of the fittest: Accelerating convergence in full configuration-interaction
quantum Monte Carlo. J. Chem. Phys..

[125] Spencer, J. pyblock; https://github.com/jsspencer/pyblock, (accessed 18 May 2026).

[126] Widmark P.-O., Malmqvist P.-Å., Roos B. O. (1990). Density matrix averaged
atomic natural orbital (ANO) basis sets for correlated molecular wave
functions. Theor. Chim. Acta.

[127] Pou-Amérigo R., Merchán M., Nebot-Gil I., Widmark P.-O., Roos B. O. (1995). Density matrix averaged
atomic natural
orbital (ANO) basis sets for correlated molecular wave functions. Theor. Chim. Acta.

[128] Angeli C., Calzado C. J. (2012). The role of the magnetic orbitals
in the calculation of the magnetic coupling constants from multireference
perturbation theory methods. J. Chem. Phys..

[129] García V., Castell O., Caballol R., Malrieu J. (1995). An iterative
difference-dedicated configuration interaction. Proposal and test
studies. Chem. Phys. Lett..

[130] Calzado C. J., Sanz J. F., Malrieu J. P. (2000). Accurate
ab initio
determination of magnetic interactions and hopping integrals in La2-xSrxCuO4
systems. J. Chem. Phys..

[131] Blunt N. S., Booth G. H., Alavi A. (2017). Density matrices
in
full configuration interaction quantum Monte Carlo: Excited states,
transition dipole moments, and parallel distribution. J. Chem. Phys..

[132] Kahn, O. Molecular Magnetism; VCH Publishers; New York, 1993.

[133] Calzado C. J., Angeli C., Taratiel D., Caballol R., Malrieu J.-P. (2009). Analysis
of the magnetic coupling in binuclear systems.
III. The role of the ligand to metal charge transfer excitations revisited. J. Chem. Phys..

[134] Oms O., Rota J.-B., Norel L., Calzado C. J., Rousseliére H., Train C., Robert V. (2010). Beyond Kahn’s
Model Substituent
and Heteroatom Influence on Exchange Interaction in a Metal-Verdazyl
Complex. Eur. J. Inorg. Chem..

[135] Rota J.-B., Calzado C. J., Train C., Robert V. (2010). Microscopic
origins of the ferromagnetic exchange coupling in oxoverdazyl-based
Cu (II) complex. J. Chem. Phys..

[136] Shuku Y., Suizu R., Domingo A., Calzado C. J., Robert V., Awaga K. (2013). Multidimensional Network
Structures
and Versatile Magnetic Properties of Intermolecular Compounds of a
Radical–Anion Ligand, [1,2,5]­Thiadiazolo­[3,4-f]­[1,10]­phenanthroline
1,1-Dioxide. Inorg. Chem..

[137] Tenti L., Maynau D., Angeli C., Calzado C. J. (2016). Highly
efficient perturbative + variational strategy based on orthogonal
valence bond theory for the evaluation of magnetic coupling constants.
Application to the trinuclear Cu­(II) site of multicopper oxidases. Phys. Chem. Chem. Phys..

[138] Giner E., Tew D. P., Garniron Y., Alavi A. (2018). Interplay
between Electronic Correlation and Metal–Ligand Delocalization
in the Spectroscopy of Transition Metal Compounds: Case Study on a
Series of Planar Cu^2+^ Complexes. J. Chem. Theory Comput..

