# Supplementary material for: Stochastic Difference-Dedicated Configuration Interaction for Magnetic Exchange in Large Active Spaces
Source: J Chem Theory Comput. 2026 Jun 16;22(13):6572–83. doi: 10.1021/acs.jctc.6c00620 (PMC13374043; doi:10.1021/acs.jctc.6c00620)
Supplement: Supplementary file 1 [file ct6c00620_si_001.pdf]

Stochastic Difference-Dedicated Configuration Interaction for Magnetic Exchange in Large Active Spaces

Luca Bonfirraro,[*,†] Oskar Weser,[†,§] Carmen J. Calzado,[‡] Vincent Robert,[¶] and Giovanni Li Manni[*,†]

†*Max Planck Institute for Solid State Research, Heisenbergstr. 1, 70569 Stuttgart, Germany*

‡*Departamento de Química Física, Universidad de Sevilla, c/Profesor García González, s/n., 41012 Sevilla, Spain*

¶*Laboratoire de Chimie Quantique de Strasbourg, Institut de Chimie de Strasbourg, CNRS/Université de Strasbourg, 4 rue Blaise Pascal, 67000 Strasbourg, France*

§*Current address: European Centre for Medium Range Weather Forecast, Robert Schuman Platz 3, 53175, Bonn*

E-mail: l.bonfirraro@fkf.mpg.de; g.limanni@fkf.mpg.de

S1 Results

Table S1: Non-stochastic absolute ground state energies, using a CAS(9,9) reference.

	$E(S)$ / Ha			
Spin S	CAS(9,9)	DDCI1	DDCI2	DDCI3
1/2	-5953.461509	-5953.471386	-5953.474075	-5953.521811
3/2	-5953.461797	-5953.471690	-5953.474383	-5953.522122
5/2	-5953.462115	-5953.471593	-5953.474207	-5953.521881
7/2	-5953.462549	-5953.471336	-5953.473822	-5953.521391
9/2	-5953.463090	-5953.470930	-5953.473249	-5953.520706

Table S2: Stochastic absolute ground state energies, using a CAS(9,9) reference.

	$E(S)$ / Ha			
Spin S	CAS(9,9)	DDCI1	DDCI2	DDCI3
1/2	-5953.461500	-5953.471386	-5953.474092	-5953.521918
3/2	-5953.461790	-5953.471680	-5953.474398	-5953.522230
5/2	-5953.462110	-5953.471590	-5953.474222	-5953.521986
7/2	-5953.462541	-5953.471337	-5953.473840	-5953.521497
9/2	-5953.463090	-5953.470930	-5953.473270	-5953.520811

Table S3: Stochastic absolute ground state energies, using a CAS(9,15) reference.

	$E(S)$ / Ha			
Spin S	CAS(9,15)	DDCI1	DDCI2	DDCI3
1/2	-5953.461646	-5953.527285	-5953.643060	-5953.710382
3/2	-5953.461911	-5953.527574	-5953.643485	-5953.710852
5/2	-5953.462203	-5953.527326	-5953.643150	-5953.710478
7/2	-5953.462602	-5953.526772	-5953.642509	-5953.709917
9/2	-5953.463096	-5953.526017	-5953.641631	-5953.709036

Table S4: Stochastic absolute ground state energies, using a CAS(33,21) reference.

	$E(S)$ / Ha				
Spin S	CAS(33,21)	DDCI1	CAs(33,21)+1h	CAS(33,21)+1h1p	CAS(33,21) + 1p
1/2	-5953.464239	-5953.585714	-5953.464660	-5953.480497	-5953.563242
3/2	-5953.464399	-5953.585979	-5953.464820	-5953.480718	-5953.563811
5/2	-5953.464589	-5953.584970	-5953.465005	-5953.480888	-5953.563014
7/2	-5953.464851	-5953.583343	-5953.465242	-5953.481109	-5953.561666
9/2	-5953.465190	-5953.581278	-5953.465553	-5953.481390	-5953.559864

S2 Stochastic-DDCI convergence

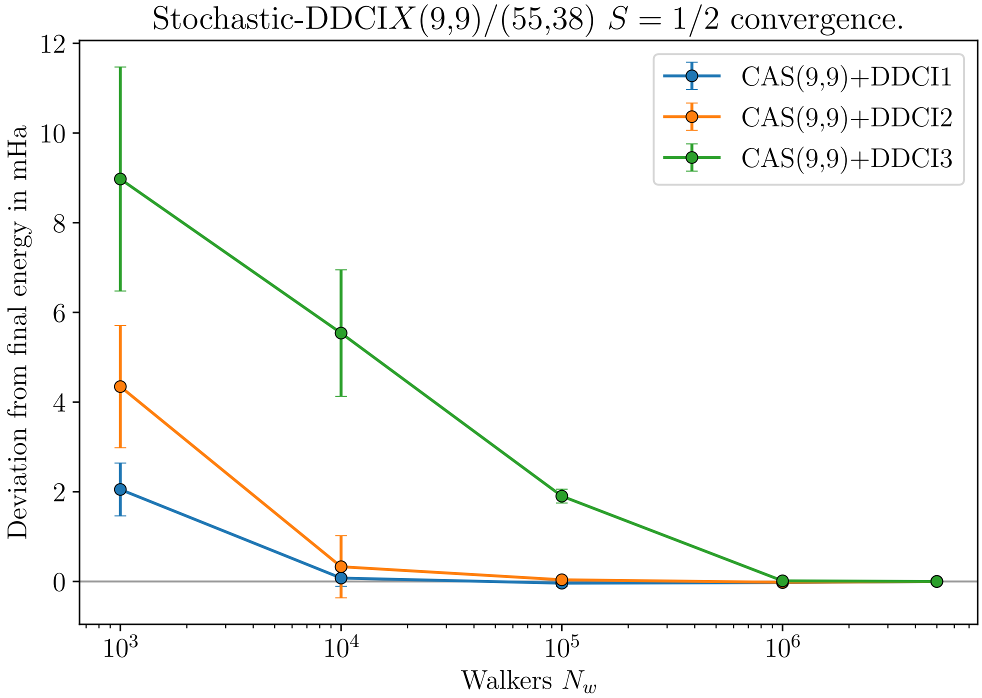

Figure S1: Convergence of Stochastic-DDCIX(9,9)//(55,38) for the $S = 1/2$ state with respect to the number of walkers.

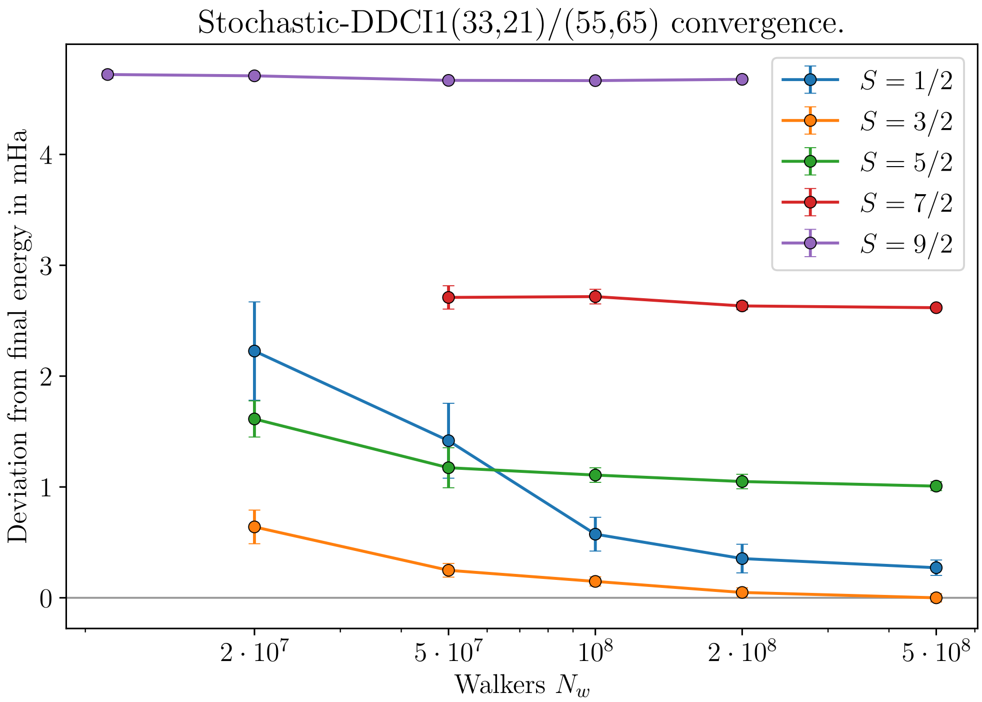

Figure S2: Convergence of Stochastic-DDCI1(33,21)//(55,65) with respect to the number of walkers.
